# What Can We Learn about Fall Risk Factors from EHR Nursing Notes? A Text Mining Study

**DOI:** 10.5334/egems.237

**Published:** 2018-09-20

**Authors:** Ragnhildur I. Bjarnadottir, Robert J. Lucero

**Affiliations:** 1 University of Florida, US

**Keywords:** text mining, nursing, patient safety, health informatics, data science

## Abstract

**Introduction:**

Hospital falls are a continuing clinical concern, with over one million falls occurring each year in the United States. Annually, hospital-acquired falls result in an estimated $34 billion in direct medical costs. Falls are considered largely preventable and, as a result, the Centers for Medicare and Medicaid Services have announced that fall-related injuries are no longer a reimbursable hospital cost. While policies and practices have been implemented to reduce falls, little sustained reduction has been achieved. Little empirical evidence supports the validity of published fall risk factors. While chart abstraction has been used to operationalize risk factors, few studies have examined registered nurses’ (RNs’) narrative notes as a source of actionable data. Therefore, the purpose of our study was to explore whether there is meaningful fall risk and prevention information in RNs’ electronic narrative notes.

**Methods:**

This study utilized a natural language processing design. Data for this study were extracted from the publicly available Medical Information Mart for Intensive Care (MIMIC-III) database. The date comprises deidentified EHR data associated with patients who stayed in critical care units between 2001 and 2012. Text mining procedures were performed on RN’s narrative notes following the traditional steps of knowledge discovery.

**Results:**

The corpus of data extracted from MIMIC-III database was comprised of 1,046,053 RNs’ notes from 36,583 unique patients. We identified 3,972 notes (0.4 percent) representing 1,789 (5 percent) patients with explicit documentation related to fall risk/prevention. Around 10 percent of the notes (103,685) from 23,025 patients mentioned intrinsic (patient-related) factors that have been theoretically associated with risk of falling. An additional 1,322 notes (0.1 percent) from 692 patients (2 percent) mentioned extrinsic risk factors, related to organizational design and environment. Moreover, 7672 notes (0.7 percent) from 2,571 patients (7 percent) included information on interventions that could theoretically impact patient falls.

**Conclusions:**

This exploratory study using a NLP approach revealed that meaningful information related to fall risk and prevention may be found in RNs’ narrative notes. In particular, RNs’ notes can contain information about clinical as well as environmental and organizational factors that could affect fall risk but are not explicitly recorded by the provider as a fall risk factors. In our study, potential fall risk factors were documented for more than half of the sample. Further research is needed to determine the predictive value of these factors.

**Implications for Policy or Practice:**

This study highlights a potentially rich but understudied source of actionable fall risk data. Furthermore, the application of novel methods to identify quality and safety measures in RNs’ notes can facilitate inclusion of RNs’ voices in patient outcomes and health services research.

## Introduction

Falls are a national public health problem across the care continuum. Hospitalization increases the risk of falls [1]. It has been estimated that between 700,000–1,000,000 falls occur in United States (U.S.) hospitals each year with associated total direct medical costs of $34 billion [1,2]. Between 30 and 50 percent of hospital-acquired falls result in injuries, and up to 10 percent of those who fall sustain serious injuries, including fractures and brain injuries [3]. Injurious falls in the hospital contribute to longer hospital stays, unnecessary readmissions and added hospital costs. Additionally, patients who suffer a fall in the hospital may experience fear of falling, mobility impairments and other factors that increase the risk of subsequent falls. Research indicates that at least one third of hospital falls are preventable [4]. Since 2008, the Centers for Medicare and Medicaid Services have stopped reimbursing hospitals for traumatic injuries resulting from a fall [5]. Consequently, the potential for cost savings are substantial if hospitals could effectively predict whether a patient is likely to fall during hospitalization. The prevalence of falls varies between different clinical settings. Falls are a rare event in critical care settings [6], which has been attributed to higher patient acuity and higher staff-to-patient ratios. However, falls occur in this setting with devastating effects to patients and families as well as unit staff. Moreover, nurses in critical care settings manage complex care and competing priorities for their patients, making the need to identify reliable and valid predictors of fall risk even more crucial [7].

While policies and practices have been implemented to reduce hospital-acquired falls, little sustained reduction has been achieved in U.S. hospitals [1,3,6]. This may be in part because existing assessment tools and prediction models used to detect patients at risk for falling have limited predictive value [8]. Moreover, existing models vary in what factors predict a fall. These inconsistencies have hindered a full understanding factors associated with the problem of hospital falls [9,10]. The most commonly identified factors associated with fall risk (e.g. age and gender) may not be modifiable by nursing interventions and factors that are actionable by nurses appear less often in fall risk prediction models. Even though evidence indicates that nursing care and organization factors can impact health outcomes [11,12], prediction models do not generally account for nursing assessment data, including organizational features (e.g., nurse staffing or skill mix) and nurse flowsheets and progress notes [13]. This presents an opportunity to discover the relative contribution of the organization and practice of nursing on fall risk.

Clinicians document important clinical information in the form of structured lists (i.e. structured data) and free text, such as nursing progress notes. It is estimated that 75 percent of available electronic health record data is in narrative form [14]. In progress notes, clinicians document additional/supplementary patient care information they consider important, but might not be captured through structured data fields. Researchers have reported on consistent patterns in nurses’ progress note documentation that can predict adverse patient outcomes, such as cardiac arrest [15,16]. Even though information contained in nurses’ progress notes is clinically important, researchers have documented that these notes are rarely read by other clinicians. Moreover, research has focused narrowly on examining the structure and content of nurses’ progress notes [17,18].

Analyses of unstructured data come with unique challenges because it is not directly computer-readable. Textual data cannot be analyzed using traditional statistics [19]. Using and identifying methods to extract and analyze nurses’ progress notes can result in generating knowledge of factors related to patients’ risk of adverse outcomes, such as falling.

Text mining has emerged as a promising method to extract and analyze clinical progress notes [17]. Research has demonstrated that text mining can be effective in identifying data from clinical progress notes, including chart review and adverse event detection [20,21,22,23]. The existing body of text mining research has focused largely on the application of text mining in physician-generated notes. Other narrative data exist to study the content and potential contribution of registered nurses (RNs)—e.g., RN-generated electronic health record (EHR) clinical progress notes [17].

Given that nursing progress notes are an understudied data source, an important first step to address is to examine the content of RN-generated EHR progress notes. This includes determining whether and to what extent content that is clinically or theoretically related to the topic of study (i.e., falls) is present in RN progress notes. Therefore, the purpose of this text mining study was to explore whether there is meaningful fall risk and prevention information in RNs’ EHR progress notes.

## Methods

This study applied text mining methods to examine content related to fall risk and prevention in RNs’ electronic narrative notes. Text mining automatically identifies features or information from free text using controlled vocabularies, rule sets, reference dictionaries or lexicons. These can either be based on existing standard vocabularies or terminologies, like the Systemized Nomenclature of Medicine-Clinical Terms (SNOMED-CT) [24], or be based on subject matter expertise. This study utilized a combination of these, as will be described in the following section.

### Theoretical framework

**Figure 1:**
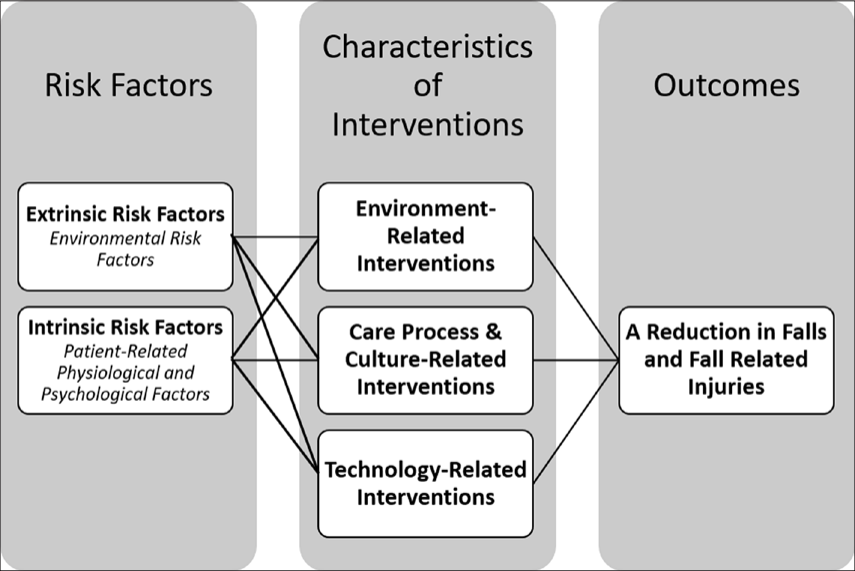
A Multi-Systemic Fall Prevention Model [25].

This text mining study was informed by a multi-systemic fall prevention model developed by Choi et al. [25]. The multi-systemic fall prevention model depicts relationships between risk factors and interventions impacting the outcome of falls and fall-related injuries. Risk factors are characterized as either intrinsic (patient-related) or extrinsic (environmental) risk factors. Interventions are characterized as either environment-related, care process or culture-related, or technology-related factors. The model is displayed in Figure 1.

### Data source and setting

This study utilized the Medical Information Mart for Intensive Care (MIMIC) III open-source dataset, maintained and shared by PhysioNet [26]. The data represents patients admitted to critical care units at a large medical center in the Northeast United States between the years 2001 and 2012. The full data set contains data from 38,597 distinct adult patients and 49,785 hospital admissions that has been deidentified using a rigorously evaluated deidentification system [26]. Sample characteristics and further information on the MIMIC III dataset have been previously reported. This study used narrative notes included in the MIMIC III dataset under the label NOTEEVENTS. The data file was further filtered to include only nursing notes. Filtering based on whether a patient in the sample had suffered a fall during their hospital admission or not was not possible as the data is unlabeled. Therefore, we applied text mining to all nursing notes of all patients in the sample to identify factors theoretically (rather than statistically) associated with the risk of falling.

### Text mining process

This study used the Java programming language [27] and the MySQL database management system [28], and followed the traditional approach of knowledge discovery in data bases [29]. This approach is comprised of five steps: 1) Data selection, 2) Pre-processing, 3) Transformation, 4) Data mining and 5) Interpretation [29]. The first step involved the selection and acquisition of MIMIC III data. The second step, preprocessing, involved the cleaning of the data and setting the data up in a Structured Query Language (SQL) database to enable data analysis. Following initial data preprocessing, the data was transformed into n-grams using a MySQL n-gram parser. An n-gram is a sequence of a certain number of words from a larger string of words, such as a sentence [19,30]. Examples of potential n-grams related to this study would be “accidental” (unigram), “fall risk” (bigram) and “fall risk assessment” (trigram). This study used a combination of unigrams, bigrams and trigrams, which has been found to yield higher accuracy in text categorization, compared to the use of only one type of n-gram [30].

For the data mining step, a preliminary lexicon of words and terms that are clinically or theoretically related to patient falls was developed. The lexicon served as a reference dictionary for text mining in this study. The lexicon was developed by first noting words and terms that emerged from a systematic review of literature on fall risk prediction modelling [13]. Second, two leading fall risk assessment tools, STRATIFY [31] and the Morse Fall Scale [32], were examined and terms from these added to the lexicon. Third, the fall prevention toolkit developed by the Agency for Healthcare Research and Quality (AHRQ) [4] was explored for any additional words or terms to add to the lexicon. Fourth, the International Classification of Diseases (ICD) 9 [33], SNOMED-CT [24], the Logical Observation Identifiers Names and Codes (LOINC) [34] and the NANDA International terminologies [35] were searched for any additional words or phrases to add to the lexicon. Finally, the content of the lexicon was discussed with subject matter experts. Subject matter expertise was defined as: 1) advanced degree in nursing or health care data analytics, 2) demonstrated record of clinical research, or 3) at least 5 years of bedside clinical experience. In total, we consulted seven subject matter experts. Two had expertise in health care data analytics, including public health and engineering and mathematics and applied statistics. The remaining five had advanced nursing degrees (i.e., master and doctoral degrees) and current clinical practice experience. Two of the five clinical experts had a record of clinical research with a focus on patient safety and quality and health services and nursing workforce. After discussions with subject matter experts, additional terms were added to the lexicon based on their expert feedback. The lexicon is displayed in Appendix 1. Based on the theoretical framework described above [25], the lexicon used in this study included words and phrases explicitly referring to fall risk, events or prevention activities, as well as patient, environmental and organizational factors that have empirically or theoretically been linked to the risk of hospital-acquired falls. This theory- and expert-derived lexicon was used as the reference dictionary for n-gram extraction. N-gram extraction was performed using MySQL to identify notes containing n-grams matching terms from the theory- and expert-derived lexicon. An iterative process was applied by examining a subset of extracted notes and adding any additional n-grams (words or terms) that were identified. Instances of note entries matching n-grams or terms from the lexicon were indexed in a new table within the SQL database to enable further exploration of the content. The final step, interpretation, involved examining the findings and determining when additional notes were no longer being identified through new text mining iterations. This was accomplished after three iterations of the text mining process [29].

## Results

The analytical sample was comprised of 1,046,053 RN-generated EHR progress notes from 36,583 unique patients. Notes containing any n-grams related to fall risk and prevention were identified for two thirds of the patients. The most frequently identified n-grams are displayed in Tables 1a, 1b and 1c.

**Table 1a:** Risk Factors: Most frequently identified n-grams and their total frequency.

Intrinsic		Extrinsic	

N-gram	Total frequency	N-gram	Total frequency

iv/intravenous	167305	risk for injury/risk of injury	480
sedative	120377	safety issue	218
follows commands	44136	staffing	100
confused	37762	low light	65
ambulation	11812	out of reach	34
not following commands	9735	long hours	27
FRID	9085	ill-fitting/ill fitting	43
dizziness	7637	hazardous	12
to get oob	6835	new nurse	7
low bp/low blood pressure	7146	clutter	5

**Table 1b:** Most frequently identified n-grams and their total frequency.

Environment-Related		Care Process & Culture-Related		Technology-Related	

N-gram	Total frequency	N-gram	Total frequency	N-gram	Total frequency

safety precautions	1302	monitor mental status	3657	bed alarm/bedalarm	4784
bed low	730	maintain safety	2512	call light/callight	1654
SR up	469	safety measures	846	chair alarm	574
arm rest	371	family education	105	electronic	158
bed rail/bedrail	327	id bracelet	38	call button	81
need for restraints	200	bed sitter	35	safety device	73
low bed	72	patient education	31	non-slip	34
equipment safe	19	medication review	29	gait belt	29
safety procedure	8	educated family	16	no slip	13
hand rails	4	educated patient	11		

**Table 1c:** Explicit mentions of fall risk, events or prevention: Most frequently identified n-grams and their total frequency.

Explicit fall risk, events or prevention	

N-gram	Total Frequency

mechanical fall	868
fall risk	837
fall precaution	718
risk for fall/risk of fall	469
fell from	320
patient fell/patient fell	265
history of fall	113
fall prevention	57
recurrent fall	24
time of fall	23

### Risk factors

The most commonly extracted n-grams were those related to intrinsic risk factors to patient falls, identified in 103,685 notes (10 percent) from 23,025 patients (63 percent). These included physiological and psychological patient factors, such as poor gait, mobility issues and impaired cognition. Additionally, 1,322 notes (0.1 percent) from 692 patients (2 percent) were found to contain n-grams related to extrinsic risk factors, such as environmental hazards and staffing and organizational features. Examples include mentions of understaffing, unstable furniture and slippery floors. In 3,972 notes (0.4 percent) from 1,789 patients (5 percent), fall risk or fall events were explicitly referenced (e.g. “Fell on slippery floor”, “Fell from bed”).

### Intervention characteristics

In terms of interventions, 7,672 records (0.7 percent) from 2,571 patients (7 percent) were identified as containing n-grams to any of the intervention characteristics. The most commonly extracted n-grams were those associated with technology-related interventions, such as bed alarms and call lights. Moreover, care process and culture interventions, including monitoring mental status and maintaining safety, were commonly identified. Additionally, environment-related interventions, including removal of environmental clutter and lowering beds and chairs were frequently referenced.

## Discussion

This exploratory study of RN-generated EHR progress notes revealed clinically meaningful information about risk factors and intervention characteristics that could be significantly associated hospitalized patient’s fall risk. Specifically, in the corpus of RN progress notes for critical care patients, notes containing n-grams matching terms from the theory- and expert-derived lexicon were identified for around two thirds of the patients. Notably, this includes information that is not traditionally captured in structured fields of patient’s records, such as staffing and environmental factors.

Existing fall-risk prediction models lack specificity and fail to out-perform clinical judgement in terms of predictive value [13]. RNs’ EHR progress notes may contain patterns of documentation and information that are not apparent in structured data. Additionally, RNs’ EHR progress notes include important context and clinical rationale that could help improve our understanding of fall risk and effective prevention. Examination of these textual data could generate hypotheses for further research on previously understudied fall risk factors. This includes hypothesis generation related to the impact of organizational design, such as hospital environment and staffing, on fall risk and prevention. Studying nurse-generated progress notes presents a unique opportunity to leverage nurses’ voices in health care improvement. Nurses’ voices have been historically underrepresented in health services and policy research [36,37,38]. The identification of statistically and clinically meaningful risk factors from nurse-generated data, as well as innovative methods, can inform the development of effective clinical nursing practice.

Text mining has been used previously to examine patient safety, including identifying adverse events, automatically classify severity of incidents, and identify falls in ambulatory settings [20,21,22,39]. This study adds to this body of evidence by highlighting the potential value of acute care nurses progress notes, a previously understudied data source. The lexicon generated in this study is both expert- and data-driven and can be adopted and/or adapted in other text mining analyses for external validation.

### Limitations

This exploratory text mining study provides an important first step in determining the potential value of RNs’ electronic narrative notes for fall prediction and prevention in hospital settings. However, several limitations should be noted. First, the data were limited to critical care patients in a single hospital system. Documentation practices may vary between different sites and settings. The supervised text mining approach constrained our results based on the lexicon created for this exploratory study. Therefore, our findings may be a conservative estimate of potentially significant and meaningful content in these RN-generated EHR progress notes. However, the lexicon development methods contributed to further generalizability and clinical meaningfulness across settings. Applying methods that included a review of the literature and consulting with subject matter experts from varied clinical and geographical backgrounds meant that terms in the lexicon should be representative of documentation in a broader range of settings and geographic regions. Second, the dataset used in this study was unlabeled. In other words, we were unable to distinguish between those who fell during their hospitalization and those who did not fall. We could only conclude that RN-generated EHR progress notes might contain risk factors that have been theoretically and empirically linked to fall risk.

## Conclusions

The findings of this study highlight a rich source of RN EHR data that has been underutilized in clinical and organizational studies of hospital adverse events. Application and development of more robust text mining methods could uncover meaningful predictors or features related to patient falls and other patient safety outcomes. Future research should focus on more robust text mining analytics, including predictive analytics and unsupervised methods, using data that contain labels that reliably describe important dependent variables (e.g., faller or non-faller) These analytics can be used to uncover previously unknown fall risk factors, account for clinical and contextual features (i.e., prevalence, time before/after fall), and evaluate the predictive value of assessment data found in RN EHR progress notes.

## Additional File

The additional file for this article can be found as follows:

10.5334/egems.237.s1 Appendix 1 Literature and expert-driven lexicon.
