# Supplementary material for: What Can We Learn about Fall Risk Factors from EHR Nursing Notes? A Text Mining Study
Source: EGEMS (Wash DC). 2018 Sep 20;6(1):21. doi: 10.5334/egems.237 (PMC6157016; doi:10.5334/egems.237)
Supplement: Appendix 1 — Literature and expert-driven lexicon. [file egems-6-1-237-s1.pdf]

Appendix 1: Literature and expert-driven lexicon

	Direct mentions of fall risk, events or prevention	Intrinsic risk factors	Extrinsic risk factors	Interventions
Unigrams	Fall	agitated	safety	
	Fell	transfer	hazard	
	trip	mobility	staffing	
	tumble	cane	turnover	
	stumble	walker	workload	
	slip	crutches		
	incident	gait		
	accident	bedrest		
		wheelchair		
		disoriented		
		overestimates		
		ambulation		
		wandering		
Bigrams	Accidental fall	visually impaired	safety hazard	frequent toileting
	near fall	transfer score	poor lighting	foot care
	fall efficacy	mobility score	insufficient lighting	environmental safety
	fall related	secondary diagnosis	unstable furniture	equipment safety
	reduce falls	overestimates limitations	unsafe flooring	hazard communication
	fall prevention	forgets limitations	high turnover	safety finding
	falls analysis	intravenous therapy	staffing patterns	safety observation
	falls data	heparin lock	staff roles	safety surveillance
	fall precautions	fall circumstances	work climate	safety devices
	patient's fall	unsafe behaviors	environmental hazard	safety behavior
	fall risk	impaired standing	involuntary turnover	safety procedure
	fall rates	impaired sitting	turnover rate	safety precautions
	fall event	impaired walking	staff conflict	
	geriatric fall	acute confusion	staff retention	

	recurrent falls	chronic confusion	physical hazard	
	fall scale		staff injury	
			unqualified staff	
			inadequately assisted	
			inadequately supervised	
Trigrams	Fall efficacy scale	history of fall	environmental safety problem	safety during transfer
	falls team leader	transfer and mobility	equipment safety problem	safety during ambulation
	interdisciplinary falls team	nurse assist crutches	reported safety problems	
	falls team meeting	high risk medication	work longer hours	
	fall incident report	low blood pressure	hazardous physical site	
	location of fall	low vision precaution	staff member ill	
	time of fall	hard to reach	staff member unwell	
	falls data trended	risk for injury	staff member fatigued	
	falls data reported	impaired bed mobility	staff member inattention	
	feedback about falls	impaired physical mobility	staff member distraction	
	Fall risk factors	impaired wheelchair mobility	unqualified nursing staff	
	documentation of fall	impaired transfer ability		
	fall risk reduction			
	fall prevention practices			
	universal fall precautions			
	patient's fall risk			
	fall risk scores			
	risk for fall			
	history of fall			
	Morse fall scale			
